# Molecular Structure and Thermodynamics of CO_2_ and Water Adsorption on Mica

**DOI:** 10.1021/acs.jpcb.5c01076

**Published:** 2025-04-24

**Authors:** Mert Aybar, Hongwei Zhang, Rui Qiao, Jingsong Huang, Bobby G. Sumpter, Bicheng Yan, Shuyu Sun

**Affiliations:** † Department of Mechanical Engineering, Virginia Tech, Blacksburg, Virginia 24061, United States; ‡ Center for Nanophase Materials Sciences, Oak Ridge National Laboratory, Oak Ridge, Tennessee 37831, United States; § Physical Science and Engineering Division, King Abdullah University of Science and Technology, Thuwal, 23955, Saudi Arabia; ∥ School of Mathematical Sciences, Tongji University, Shanghai 200092, China

## Abstract

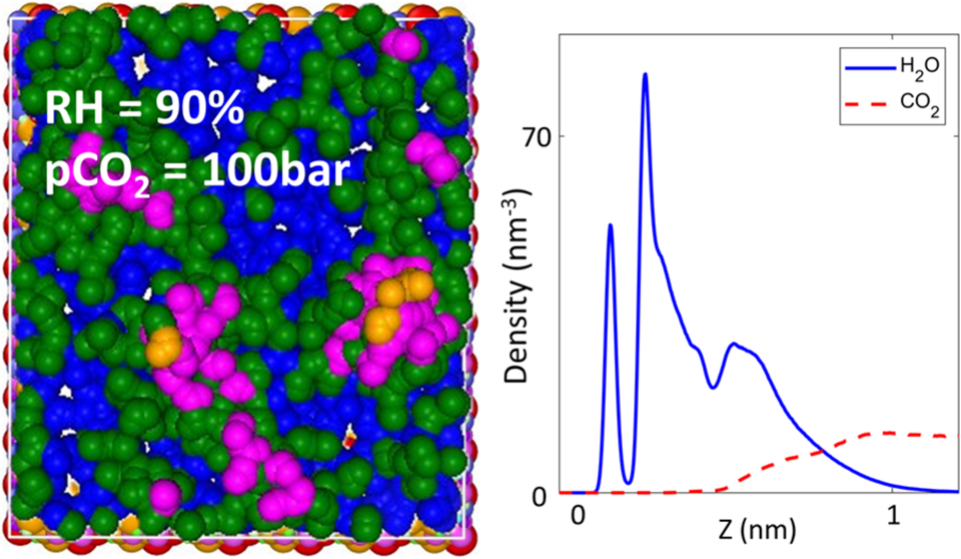

The adsorption of CO_2_ and water on clay surfaces plays a key role in applications, such as gas storage in saline aquifers and depleted hydrocarbon reservoirs, but is not yet fully understood. Here, we study the adsorption of CO_2_ and water vapor using Grand Canonical Monte Carlo and molecular dynamics simulations. At a bulk pressure of 100 bar, pure CO_2_ adsorbs strongly on mica and forms extensive layers next to it. CO_2_ adsorption is lowered substantially if introducing water vapor above mica and is largely eliminated when the relative humidity (RH) approaches about 60%. When pure water vapor is introduced above a mica surface, a subnanometer thick liquid water film develops on it to form apparent liquid–solid and liquid–vapor interfaces simultaneously. Using the identification of truly interfacial molecules (ITIM) analysis, we delineate how individual water layers develop in this film as RH increases. We highlight that the water film is spatially heterogeneous and the true liquid–vapor interface emerges only at an RH of 60–80%. Introducing 100 bar of CO_2_ into the water vapor above the mica surface modulates water adsorption nonlinearly: at RH = 0.01%, the water adsorption is reduced by ∼30%; as RH increases, the reduction is weakened, and eventually, enhancement of water adsorption by about 7% occurs at RH = 90%. These variations are attributed to the interplay of film thinning by high-pressure CO_2_, competition of mica surface sites by CO_2_ molecules, and energetic and entropic stabilization of interfacial water by CO_2_ molecules.

## 1 Introduction

The adsorption of CO_2_ and water on minerals under unsaturated conditions plays an important role in many subsurface applications. For example, the adsorption of CO_2_ molecules on mineral surfaces can displace heavy hydrocarbons and facilitate their transport through nanoscale pores, both of which improve the recovery of oil from unconventional reservoirs.^1−3^ In geological CO_2_ sequestration, water readily adsorbs on highly hygroscopic minerals to form nanometer-thin water films, and the adsorption of CO_2_ on mineral surfaces under such conditions is often a key step in their reactions and subsequent precipitation.^4−6^ Likewise, in underground hydrogen storage, water vapor can adsorb onto clay surfaces in the caprock to affect their swelling and mechanical integrity.^7−9^ The presence of CO_2_, which is sometimes used as cushion gas in such systems, can perturb the adsorption of water and consequently the performance of caprock in preventing hydrogen leakage.^7^

The adsorption of CO_2_ and water on minerals has been studied by using many methods. Among them, Grand Canonical Monte Carlo (GCMC) and molecular dynamics (MD) simulations have been extensively used due to their ability to resolve the adsorption details at the molecular level.^10−14^ For CO_2_, simulations revealed that CO_2_ can adsorb strongly on the surface of minerals, such as calcite, quartz, and montmorillonite, and surface saturation can occur at low bulk CO_2_ densities.^15−21^ The adsorbed CO_2_ molecules can easily displace not only light hydrocarbons, such as CH_4_, but also heavy carbons, such as decane and nonadecane.^22−24^

For water, Malani and Ayappa studied the adsorption of water on mica surfaces at relative humidity (RH) ranging from 0.01 to 98% using GCMC.^25^ Their computed adsorption isotherm and water film thickness agree well with the experimental measurements.^26^ As RH increases from 0 to 70%, there is a notable redistribution of water molecules between the adsorbed layers. The water molecules in the first density peak near the mica surface are strongly bound to mica; those in the second density peak, however, exhibit less solid-like structures. Later, Cheng and Sun showed that the adsorbed water molecules within 0.6 nm from the mica surface exhibit strong orientational ordering.^27^ They further concluded that the water molecules in the first two water density peaks belong to the first water monolayer adsorbed on the mica.

Previous simulations have advanced our understanding of the adsorption of CO_2_ and water on minerals under unsaturated conditions. However, many questions remain open. For example, while the development of water films on minerals with RH has been studied, most analyses were based on laterally and temporally averaged density profiles. In particular, the three-dimensional (3D), instantaneous structure of the water film and its evolution with RH are not yet known. At a more fundamental level, whenever a water film develops on a mineral surface, water–solid and water–vapor interfaces form. However, because of the solid-like structure of the innermost adsorption layer, it is unclear under what conditions (e.g., RH or water film thickness) the water–vapor interface truly becomes a liquid–vapor interface and what impact it brings. For CO_2_, previous works revealed that it is less competitive in adsorbing on kaolinite surfaces than water.^28^ However, the coadsorption of water and CO_2_ on mineral surfaces received scant attention so far.^29,30^ How the RH in the environment quantitatively affects CO_2_ adsorption, and if CO_2_ adsorption still exists, how CO_2_ molecules exist on the surface is not yet well understood. Further, how the presence of CO_2_, especially at pressure relevant to subsurface applications, affects the adsorption of water on mineral surfaces has received little attention, let alone the underlying mechanisms.

In this work, we investigate the adsorption of pure CO_2_, pure water vapor, and a CO_2_–water vapor mixture on mica surfaces using molecular simulations. The rest of the paper is organized as follows. Section 2 presents the simulation system and methods. In Section 3, the adsorption of pure CO_2_ and water vapor is discussed first, followed by the coadsorption of CO_2_ and water. The adsorption isotherms and structure of adsorbates are quantified, and the underlying mechanisms are analyzed. Finally, conclusions are drawn in Section 4.

## 2 Simulation Systems, Models, and Methods

### 2.1 Molecular Systems

**Figure 1:**
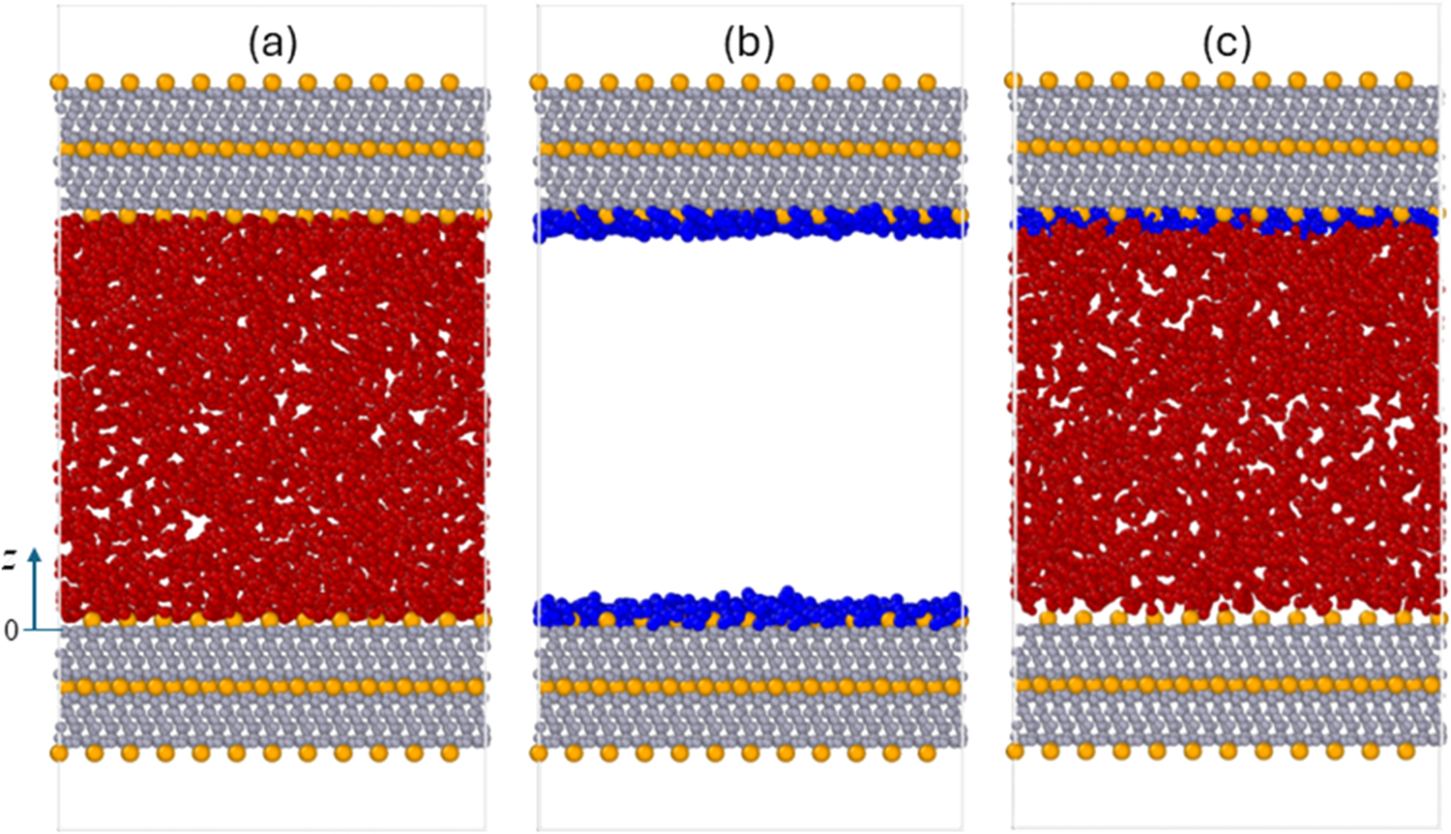
Snapshots of molecular systems for studying the adsorption of pure CO_2_ (a), pure water (b), and a CO_2_ + water vapor mixture (c) on mica. The origin of the *z* axis (*z* = 0) corresponds to the surface oxygen plane of the lower mica wall. In (c), to better delineate to what extent the CO_2_ molecules can approach mica (in particular, its surface K atoms), the water molecules within 0.6 nm of the lower mica surface are not shown. Water and CO_2_ molecules are shown using blue and red ball-and-stick models. In mica, the K atoms are shown as yellow balls, and all other atoms are shown as gray balls.

As shown in Figure 1a–c, systems A, B, and C are adopted to study the adsorption of CO_2_, water vapor, and CO_2_–water vapor mixture on mica (KAl_2_(AlSi_3_O_10_)(OH)_2_) surfaces. Each system consists of two 2 nm-thick mica slabs and the specific fluids confined between them. The mica slabs measure 6.294 nm × 5.508 nm in the *x*- and *y*-directions, respectively. The separation between the surface K^+^ ions of the two mica walls is 6 nm, so the adsorption on one wall is independent of the other. Both mica walls are frozen during the simulations. A vacuum of 1.1 nm height is placed above the top mica slab and below the bottom mica slab. Periodic boundary conditions are applied in the lateral (*x–y*) plane; the system’s periodicity in the *z*-direction is removed (see below).

The target pressure in systems A and C is controlled at 100 bar, which is relevant to the condition for CO_2_ and H_2_ underground storage, where pressure up to 200 bar can be encountered.^5,7,8^ The partial pressure of water vapor in systems B and C is controlled at 10^–4^, 10^–2^, 0.1, 0.2, 0.4, 0.6, 0.8, and 0.9 times water’s saturation pressure (*P*_0_) at our target temperature, thus producing a relative humidity (RH) in the range of 0.01 to 90%. *P*_0_ is set to 0.044 bar based on the water model we adopted and in accordance with prior GCMC studies.^25,27,31^

### 2.2 Molecular Models

The rigid SPC model^32^ is used for water molecules, and the CLAYFF force field^33^ is selected for the mica substrate. Reactions of CO_2_ are not considered. Previous simulations showed that these force fields together allow an accurate prediction of water adsorption on mica.^25,27^ We adopt the force field developed by Zhu et al.^34^ for CO_2_ molecules because it captures the thermodynamic properties of CO_2_ near the critical point and has been used extensively in the studies of CO_2_ adsorption on mineral surfaces.^15,16,18^

### 2.3 Simulation Methods

All simulations are performed using the LAMMPS code.^35^ In systems A and B, the GCMC approach is used to control the pressure of fluids in the mica channel. In system C, the GCMC approach is used to control the partial pressure of water vapor, but the CO_2_ pressure is tuned to within 5% of the target pressure (100 bar) by adjusting the number of CO_2_ molecules in the system through trial and error. Specifically, we compute the pressure of CO_2_ by measuring the force acting on each mica slab and then add/remove CO_2_ molecules from the system as needed to reach our target pressure. Note that for system C, this approach is not exact because the contribution of water to pressure is included in the measured force and pressure. However, because water’s partial pressure (at most 0.04 bar even at RH = 90%) is very low compared to the CO_2_ pressure (100 bar), the error caused by water is negligible. GCMC runs are performed using the “fix gcmc” command in LAMMPS. In these runs, GCMC and MC operations are invoked every 100 MD steps, where an average of 10 exchange attempts with an equal probability of molecule translations or rotations are performed. Convergence is typically achieved in 15 ns, but extended runs of 30 ns are required for RH = 80 and 90% to ensure good convergence.

For each system, after the number of molecules in the system reaches equilibrium through GCMC runs, a separate system is set up with the average number of molecules measured in the GCMC runs. MD simulations are then performed using this system for 4 ns to gather statistics, such as density profiles and molecule orientations.

For all systems, the temperature of the fluids is maintained at 298.15 K with a Nose–Hoover thermostat. The nonelectrostatic interactions are computed with a cutoff length of 1.2 nm. Electrostatic interactions are handled using the Particle–Particle Particle–Mesh (PPPM) method, with a real-space cutoff length of 1.2 nm and a relative accuracy of 10^–4^. The slab correction is applied in the electrostatic calculations to effectively remove the system’s periodicity in the *z*-direction. All simulations are conducted with a time step of 1 fs.

### 2.4 Interfacial Structure Analysis

**Figure 2:**
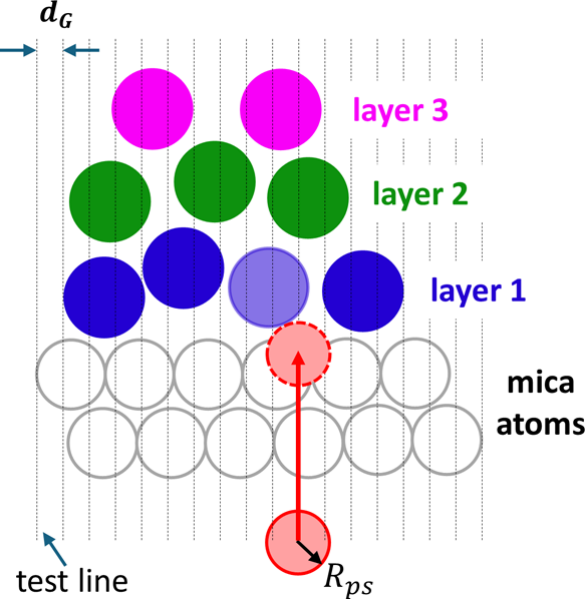
A schematic of the determination of individual molecular layers adsorbed on a solid surface using the Identification of Truly Interfacial Molecules (ITIM) methodology.

The one-dimensional (1D) number density profiles of CO_2_ and H_2_O molecules are computed across the mica channel by using the binning method in which a time-averaged histogram of molecules is obtained based on their *z*-position. Averaging over time and the lateral (*xy*-) dimensions produces a signal that can be easily compared between different systems, but doing so obscures details about the *instantaneous* and three-dimensional structures of the water film. To alleviate this limitation, we adopt the identification of the truly interfacial molecules (ITIM) analysis for several of the systems studied here to complement the 1D density profiles. An ITIM analysis seeks to reveal the structure of interfacial fluids by identifying successive layers of molecules at interfaces at each time instant.^36−38^ As shown in Figure 2, for the flat interface considered here, probing spheres (radius: *R*_ps_) are moved from a bulk phase toward the interface along test lines normal to the interface until they touch atoms of interfacial fluids. The test lines are arranged into a grid (spacing: *d*_G_). Once a probing sphere touches the first atom on its path (e.g., the light blue circle in Figure 2), the molecule to which the atom belongs is marked to be at the interface, and the method proceeds with moving the next probing sphere. Once the probing of the interface is completed, molecules identified as being at the interface (e.g., the blue circles in Figure 2) are tagged as the first interfacial layer and deleted from the system. The process is then repeated to identify the next molecular layers near the interface (e.g., the green and magenta circles in Figure 2). The above process offers a quantitative way to identify interfacial molecules. It is a powerful supplement to the 1D density profile and graphic rendering of snapshots of molecular systems widely used in literature. Because the process can be applied to many trajectory frames, ITIM also allows the number of water molecules in each interfacial layer to be averaged over many frames to improve statistics.

In this work, we use the open-source package PyTim,^39^ which leverages the MD trajectory analysis package MDAnalysis,^40^ to perform the ITIM analysis. A probing sphere radius of 0.125 nm and a grid spacing of 0.05 nm, values recommended for interfacial water by Jorge et al.,^41^ are used to identify interfacial molecules. Near the lower mica wall, probing spheres are moved from below the mica upward, and mica atoms are discarded in the ITIM process.

## 3 Results and Discussion

### 3.1 Adsorption of Pure CO_2_ and Water on Mica Surfaces

#### 3.1.1 Pure CO_2_

**Figure 3:**
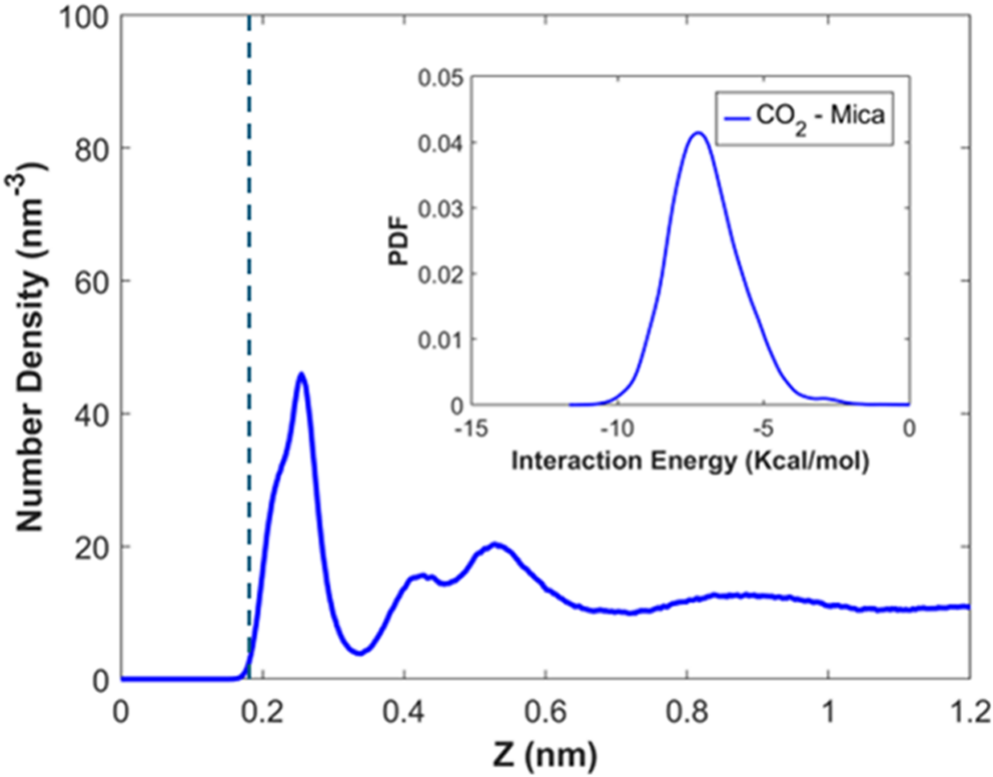
Density profile of CO_2_ near the lower mica wall when the CO_2_ pressure is 100 bar. The vertical dashed line marks the position of the surface K^+^ ions. The inset shows the distribution of the CO_2_–mica interaction energy for CO_2_ molecules in the first density peak. A CO_2_ molecule’s position is computed based on its carbon atom.

Figure 3 shows the density profile of CO_2_ normal to the lower mica wall (density near the upper wall is not shown due to symmetry). Distinct layering, with three peaks centered at *z* = 0.26, 0.42, and 0.53 nm, is observed. Such layering is consistent with the structure of dense fluids near solid surfaces.^42^ The first peak corresponds to the contact adsorption of CO_2_ on mica’s surface oxygen plane, and due to CO_2_ molecules’ relatively large size, it is located above the surface K^+^ ions. The second peak corresponds to CO_2_ molecules coordinating the surface K^+^ ions and the CO_2_ molecules in the first density peak. The third peak, poorly differentiated from the second peak, represents the CO_2_ molecules in contact with the CO_2_ molecules in the first two peaks.

The significant first peak indicates the strong adsorption of CO_2_ molecules on the mica surface. In fact, such strong adsorption is driven mainly by the electrostatic interactions between mica’s surface atoms and the CO_2_ molecules. On the one hand, while CO_2_ molecules are neutral and apolar, they have a large quadrupole moment (13.67 × 10^–40^ C·m^2^).^43^ On the other hand, the mica surface has a strong ionic nature, and its atoms carry large partial charges. Consequently, the charge–quadrupole interactions between the CO_2_ molecules and mica surface atoms are strong. Such interactions are not screened by dielectric fluids in the dry environment considered here and thus lead to a strong affinity of the CO_2_ molecules for the mica surface. Indeed, the inset in Figure 3 shows that the CO_2_–mica interactions for the CO_2_ molecules in the first peak can reach as much as −10 kcal/mol, which explains their strong adsorption (see the Supporting Information for the method for computing the interaction energy).

#### 3.1.2 Pure Water

**Figure 4:**
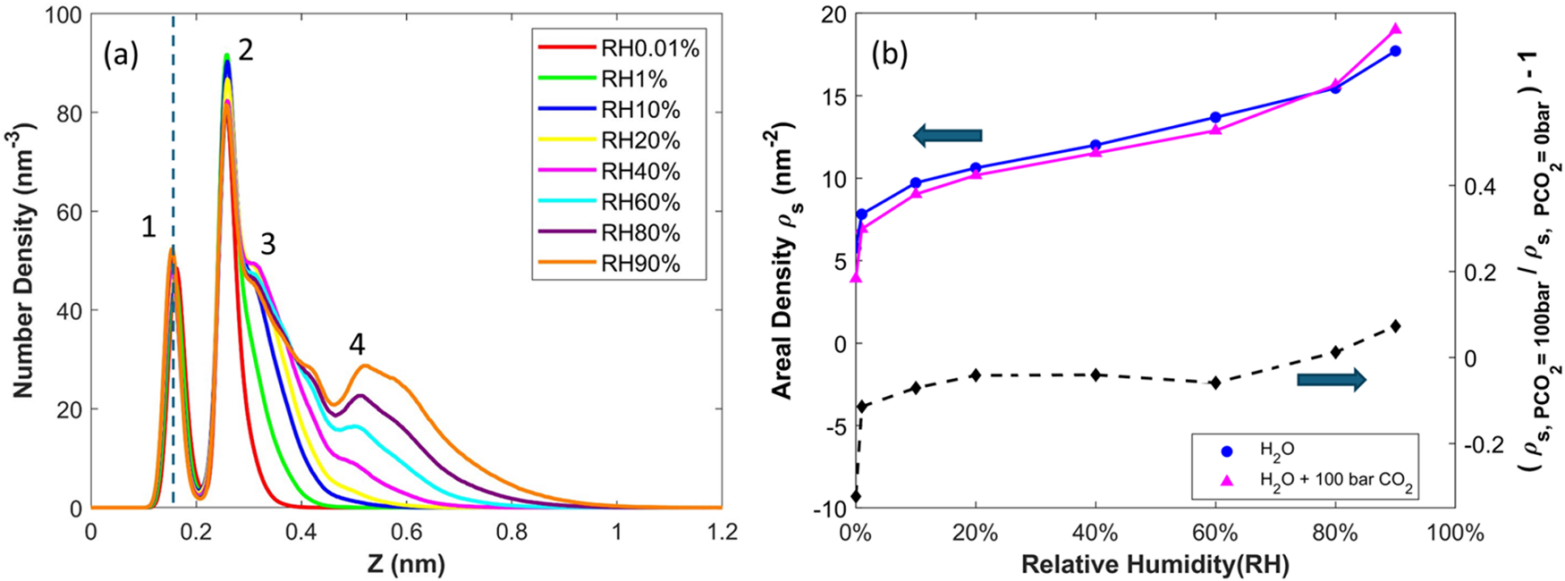
(a) Density profiles of pure water near the lower mica wall when the relative humidity (RH) in the mica channel is varied. The positions of water molecules are computed based on their oxygen atoms. (b) The adsorption isotherms of water on mica in the absence and presence of a 100 bar of CO_2_ atmosphere. In (a), 1, 2, 3, and 4 mark the four main density peaks. Water density profiles in (a) are also shown in separate panels for different RHs in Figure S1 of the Supporting Information.

Figure 4a shows the density profiles of water near the lower mica wall (*z* < 1.2 nm) when the RH in the channel is varied between 0.01 and 90%. Figure 4b shows the corresponding water adsorption isotherm, with the adsorption computed by the integration of the water density profile from the mica’s surface oxygen plane to *z* = 1.2 nm. These results closely follow those reported in previous molecular simulations^25,27^ (e.g., the isotherm at the RHs considered agrees with that reported earlier within an average difference of 3%), which agrees favorably with experimental data.^26^ Therefore, below we highlight only their salient features briefly.

At RH = 0.01%, water already adsorbs onto the mica surface to form two density peaks. The water molecules in the inner peak form hydrogen bonds with the basal oxygen atoms, while those in the second peak also hydrate the surface K^+^ ions. As RH increases, the second density peak grows and the third density peak begins to develop at RH ≈ 10%. The adsorption grows rapidly with RH up to 10%, with the increase of adsorption slowing down as RH increases. As RH increases beyond 10%, the third peak grows, which is accompanied by the redistribution of water molecules between the second and third density peaks. The adsorption grows slowly until RH reaches 60–80%. At RH beyond 60–80%, the third water density peak becomes fully developed, and a clear fourth peak emerges; this is accompanied by the rapid growth of adsorption with RH.

**Figure 5:**
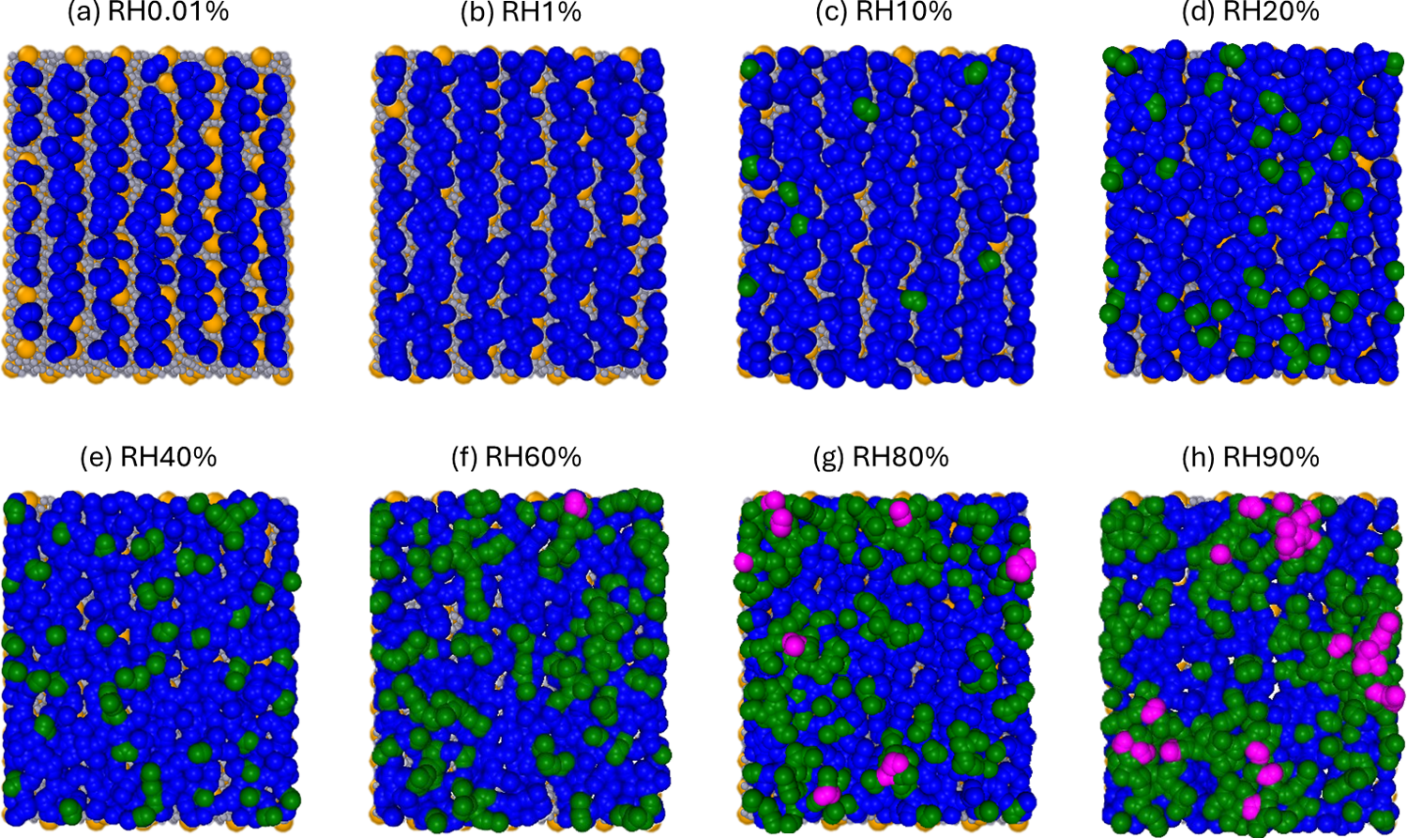
(a–h) Representative snapshots of individual water layers atop mica surfaces with RH of 0.01 to 90% in the top view. The first layer, in contact with the mica wall, is colored blue. The second (third) water layer, positioned above the first (second) layer, is colored green (magenta). The surface K^+^ ions are colored yellow.

While the 1D density profiles in Figure 4a provide some information on the development of the nanometer-thick water film on the mica surface as the environmental RH increases, it offers little insight into the instantaneous, three-dimensional (3D) structure of the thin water film. To address this limitation, we scrutinize the development of individual water layers on the mica surface as RH increases using the ITIM methodology. Figure 5a–h shows representative, top-view snapshots of individual water molecular layers atop the mica surfaces at RH of 0.01 to 90%.

At RH = 0.01%, the first water layer forms to mainly hydrate mica’s surface oxygens and lies in the interstitial space between surface K^+^ ions (Figure 5a), consistent with earlier findings.^25,27^ As RH increases to 1%, more water molecules appear near the surface K^+^ ions (Figure 5b). Although these water molecules correspond to the second peak near the mica surface shown in Figure 4a, they belong to the first water layer, because there are no water molecules beneath them. As RH increases to 10%, the first water layer densifies further, and a second water layer also emerges (Figure 5c). At this stage, water molecules in the vapor phase “see” a heterogeneous surface featuring (1) a water layer in direct contact with the mica surface (cf. blue spheres in Figures 5 and 2) and a second water layer (cf. green spheres in Figure 5c) on the top of the first water layer. Water molecules in the second layer form a coordination structure with those in the first layer but hardly associate with each other. The second water layer grows as RH increases (Figure 5c–e). When RH reaches 60–80%, the second water layer covers up most of the first water layer (cf. Figure 5f,g), and molecules in them form contacts extensively.

At RH = 80%, a third layer featuring water molecules distributed sparsely over the second water layer appears (Figure 5g). Water molecules in this third layer are coordinated with molecules in only the second water layer. As RH increases further to 90%, water molecules in the vapor phase mainly “see” the second water layer (Figure 5h). Nevertheless, at any time instant, a notable fraction of the mica surface is still covered only by a single layer of water molecules, a fact difficult to discern from the 1D density profiles in Figure 4a.

**Figure 6:**
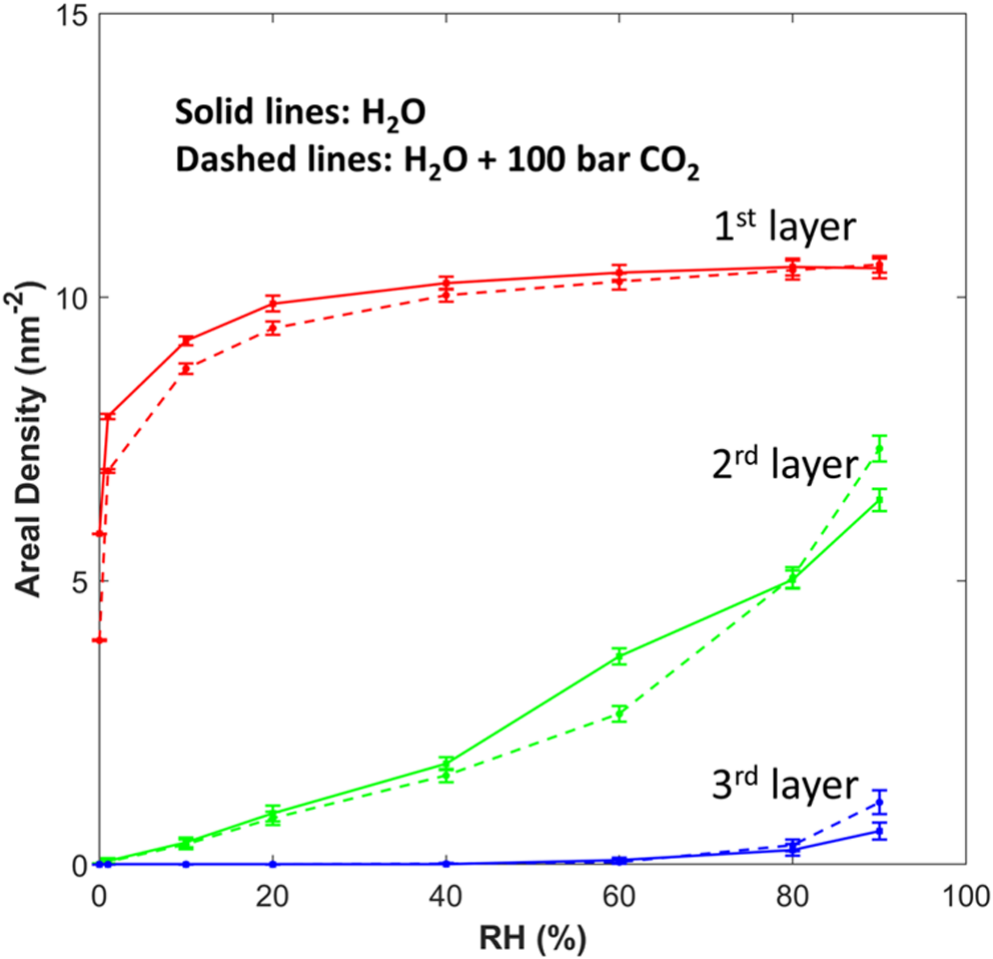
Evolution of the areal density of the first, second, and third water layers atop the mica surface as a function of the environmental RH.

Figure 6 shows the evolution of the areal density of water molecules in different layers as the RH increases. The first water layer is well formed at RH = 20%. The second water layer still grows significantly at RH = 90%, while the third water layer emerges at an RH of about 80%. Therefore, the growth of the thin water film at an elevated RH occurs through the concurrent development of the second and third water layers. At RH = 90%, the areal density of the second water layer is about 70% of that of the first water layer, suggesting that some patches of the mica surface are still covered by a single layer of water molecules, which is consistent with the snapshot in Figure 5h.

Overall, Figures 5 and 6 reveal that the water film on the top of a mica surface has a highly heterogeneous structure characterized by a different number of water molecule layers at different lateral positions. The structure revealed here and its evolution as a function of RH can have many ramifications. In the region where a mica surface is covered only by the first water layer, the water film is highly structured due to the strong water–mica interactions and distinct orientational ordering of water molecules (Figure S2). Further, water molecules in the first density peak exhibit sluggish dynamics (see Figure S3), similar to that reported near mica walls enclosing bulk water.^44^ Therefore, the water film behaves more like a solid (rather than a liquid) toward vapor phase molecules impinging on it. In regions where a mica surface is covered by an extensive second and even third water layer, in which water molecules of the same layer percolate well, molecules impinging toward the mica surface “see” a liquid-like boundary. It follows that, although a solid–water and a water–vapor interface form once a water film forms on the mica surface, the true liquid–vapor interface emerges only when the water film becomes well developed, e.g., at RH = 60–80% according to Figure 5f,g. The coverage of a mica surface by how many layers of water molecules and the emergence of a true liquid–vapor interface control the interfacial environment for other molecules (e.g., CO_2_ molecules) and consequently can affect their adsorption and geochemical reactions.

### 3.2 Coadsorption of CO_2_ and Water on Mica Surfaces

#### 3.2.1 Qualitative Features

**Figure 7:**
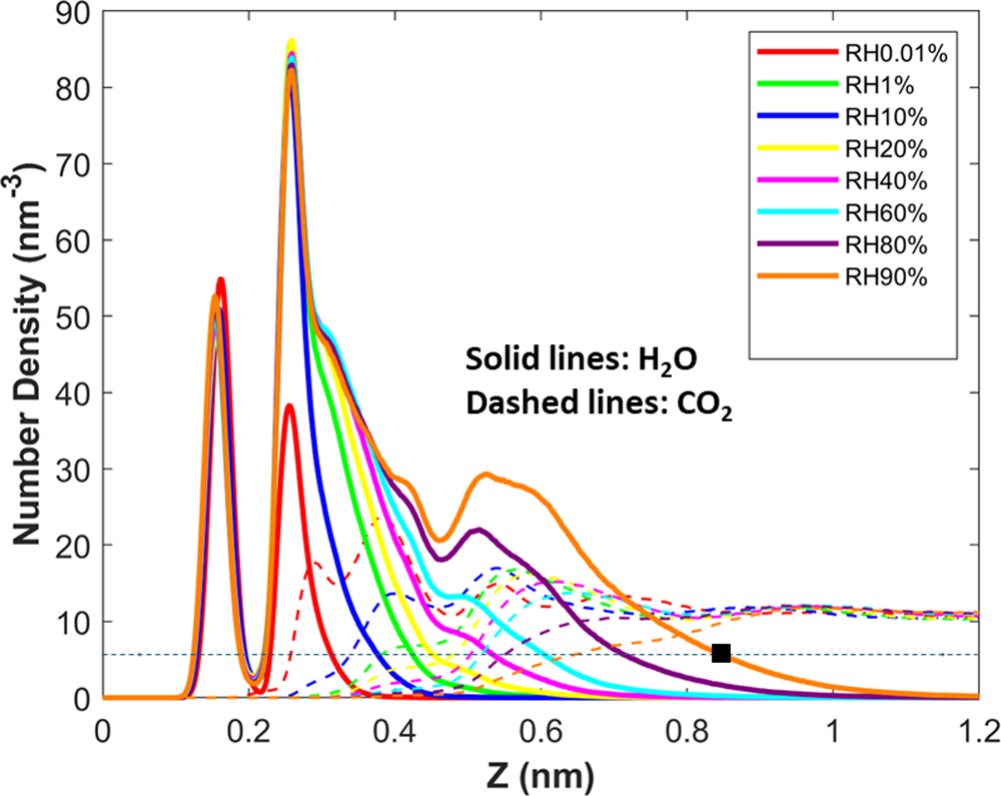
Density profiles of CO_2_ and water near the lower mica wall when the CO_2_ environmental pressure is kept at 100 bar, while RH is varied from 0.01 to 90%. Density profiles are also shown in separate panels for different RHs in Figure S1 of the Supporting Information.

Figure 7 shows the density profiles of CO_2_ and water near the lower mica wall when the CO_2_ pressure is fixed at 100 bar, while the RH is varied from 0.01 to 90%. The water density profiles qualitatively resemble those for the pure water case (Figure 4), suggesting that environmental CO_2_, even at a pressure of 100 bar, affects the water adsorption rather modestly. The CO_2_ density profiles, however, differ greatly from those of the pure CO_2_ case (cf. Figure 3). At RH = 0.01%, the first CO_2_ peak is lowered from the pure CO_2_ case, although layering, evident from the second and third peaks, remains distinct. As RH increases, the first CO_2_ peak is shifted away from the mica surface, and its height reduces further. CO_2_ layering, a key signature of solid–fluid interfaces, is maintained up to an RH of 40–60%. This trend is consistent with the fact that the water film presents a solid–vapor-like interface toward CO_2_ molecules above it up to these RHs, a key conclusion from the ITIM analysis presented above. As the RH increases beyond 60%, the CO_2_ density decreases smoothly toward the mica surface, which is consistent with the emergence of a liquid–vapor interface above the mica surface at these RHs.

#### 3.2.2 Quantitative Aspects of CO_2_ Adsorption

**Figure 8:**
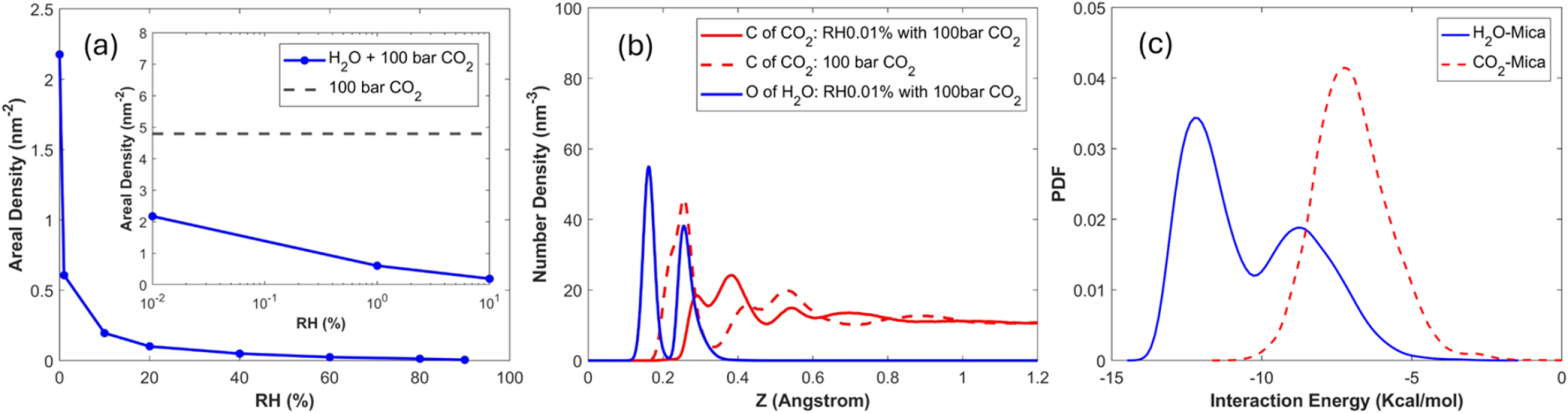
(a) CO_2_ adsorption isotherm as a function of the RH. (b) A comparison of the CO_2_ density profile near the lower mica wall when the RH is 0 and 0.01%. (c) Distribution of the interaction energy of adsorbed CO_2_ (*p*_CO_2__ = 100 bar, RH = 0) and water (*p*_CO_2__ = 0; RH = 0.01%) molecules with the mica wall.

We first quantify the contact adsorption of CO_2_ in the presence of water by integrating the CO_2_ density from *z* = 0 to *z* = 0.46 nm. The latter position corresponds to the second valley of the CO_2_ density profiles at RH = 0.01 and 1%, where CO_2_ molecules are still in contact with the mica surface and there are few water molecules, if any, beneath them. Figure 8a shows that the level of CO_2_ adsorption is reduced by about 55% when water vapor with an RH of 0.01% is introduced above the mica surface. CO_2_ contact adsorption is suppressed further as RH increases. At RH = 90%, when the water film is confined within 1 nm from the mica surface, the CO_2_ contact adsorption is reduced by about 99%.

A detailed picture of the suppressed CO_2_ adsorption at low RHs can be obtained from the CO_2_ and water density profiles at RH = 0.01%. Figure 8b shows that the thin water film pushes CO_2_ away from the mica but does not eliminate CO_2_ adsorption. In fact, two clear contact adsorption peaks centered at *z* = 0.29 and 0.38 nm are still visible. The reduced CO_2_ adsorption is attributed to the displacement by adsorbed water molecules, which is in turn caused by the stronger water–mica than the CO_2_–mica interactions. Figure 8c compares the distribution of the interaction energy for CO_2_ molecules in the first adsorption peak in system A (i.e., no water in the system) and the interaction energy for water molecules in system B (i.e., no CO_2_ in the system) at RH = 0.01% (see the Supporting Information for the calculation of these energies). The water–mica interactions are generally much stronger than the CO_2_–mica interactions because water molecules are smaller and highly polar, thereby allowing water molecules to displace CO_2_ molecules from favorable spots on the mica surface. However, the partial overlap between the CO_2_–mica and water–mica interaction energy distributions reveals that CO_2_–mica interactions are also strong, thereby explaining why CO_2_ molecules are not fully displaced from the mica surface at RH = 0.01%.

**Figure 9:**
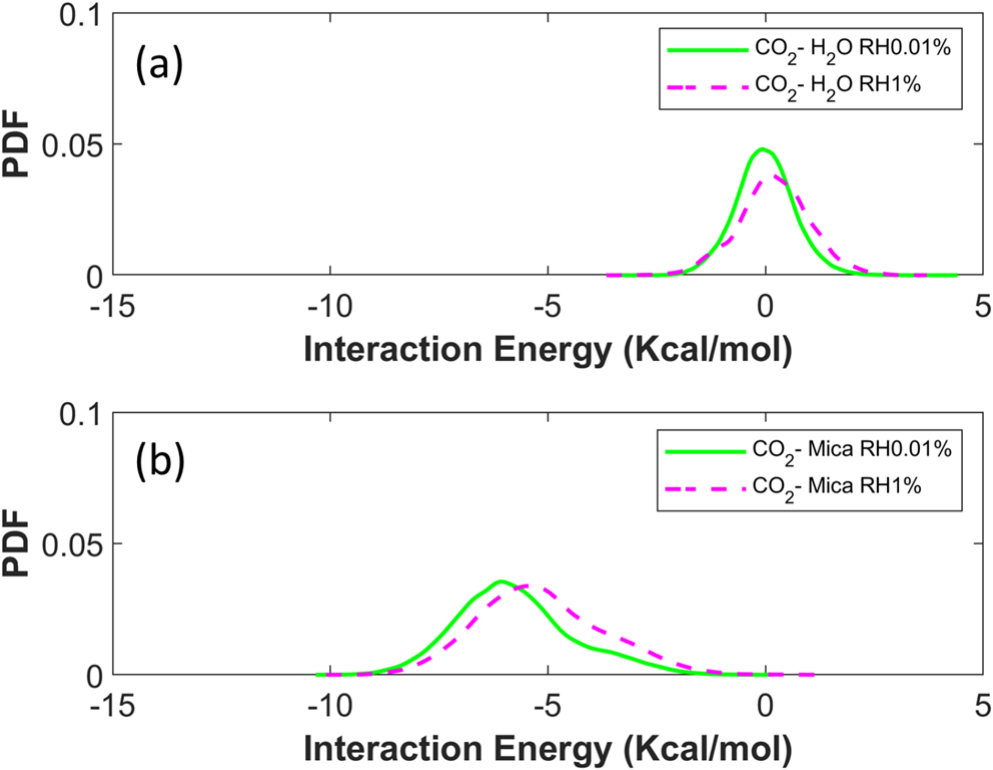
Distribution of the interaction energy of contact-adsorbed CO_2_ molecule (*z* < 0.32 nm) with mica and water at RH of 0.01% and 1%. (a) CO_2_-water interactions. (b) CO_2_–mica interactions.

Another factor that may contribute to the weakened adsorption of CO_2_ molecules is the dielectric screening of electrostatic CO_2_–mica attractions by water. The direct assessment of this screening effect is difficult, but we can infer its role by quantifying the interactions of the CO_2_–mica and CO_2_–water at different RHs. Figure 9a,b shows these interaction energies of CO_2_ molecules located below *z* = 0.32 nm at RH = 0.01 and 1%. We observe that, as RH increases from 0.01 to 1% and thus more water adsorbs on the mica (cf. Figure 4b), the CO_2_–water interaction becomes generally more positive, and its mean value shifts by about +0.21 kcal/mol, lending support to the relevance of dielectric screening of CO_2_–mica interactions. However, over the same RH window, the CO_2_–mica interaction energy also becomes more positive and its mean value shifts by about +0.59 kcal/mol. This suggests that CO_2_ molecules are displaced from their favorable spots on the mica surface, and the larger magnitude of CO_2_–mica interaction energy than the CO_2_–water interaction energy shift shows that the dielectric screening effect is relatively minor. The latter is attributed to the fact that at the low RH considered here, the dipole orientation of water molecules adsorbed on the mica surface is dominated by water–mica interactions.

**Figure 10:**
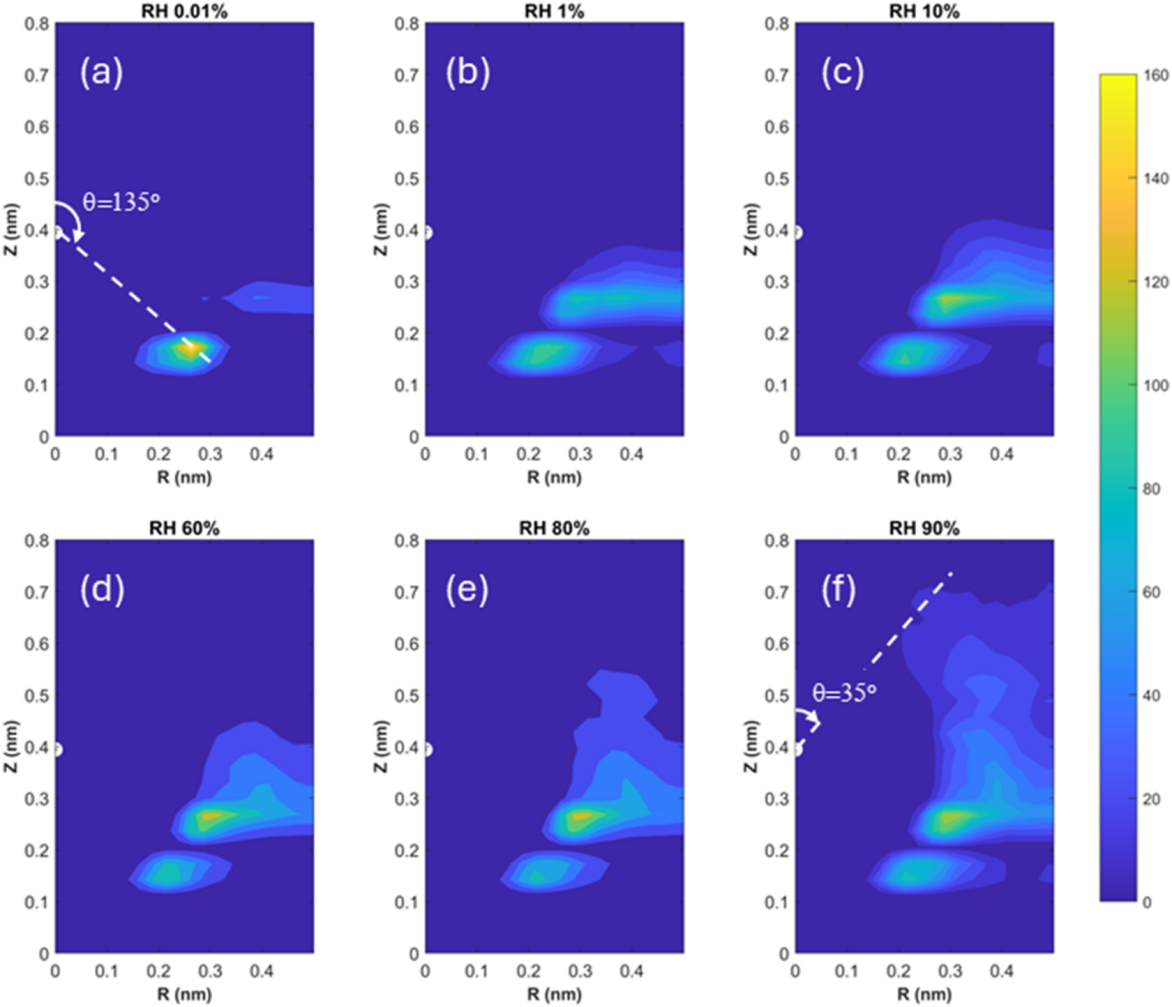
Density distribution of water molecules around CO_2_ molecules positioned at 0.4 nm above the mica surface at an RH of 0.01 to 90%. The density is plotted as a function of the water molecules’ *z*-position (*Z*) and their distance from the CO_2_ molecule’s C atom (cf. the filled white semicircle) in the *xy*-plane (i.e., the lateral direction, *R*). The CO_2_ environmental pressure is 100 bar.

The results presented above show that, in the presence of water vapor in the environment, small but nonzero contact adsorption of CO_2_ molecules can occur on the mica surface. Here, we examine the hydration environment of the contact-adsorbed CO_2_ molecules, which can potentially affect the dynamics and chemical reactivity of those CO_2_ molecules. Figure 10 shows the distribution of water around a CO_2_ molecule contact adsorbed at *z* = 0.4 ± 0.04 nm, where a CO_2_ density peak is observed at RH = 0.01 and 1% (Figure 7). Specifically, the density of water molecules is shown as a function of their *z*-position (*Z*) and distance from the CO_2_ molecule’s C atom (cf. the filled white semicircle) in the *xy*-plane (or the lateral direction, *R*). At RH = 0.01%, the CO_2_ molecule is mainly hydrated by a layer of water around a solid angle θ of 135° (the solid angle is defined relative to the CO_2_ molecules’ C atom in the (*R*, *Z*) space, see Figure 10). As RH increases (Figure 10b,c), a second layer of hydration water appears just beneath the CO_2_ molecule’s equator. As RH increases beyond 60%, additional hydration water is introduced above the CO_2_ molecule’s equator (Figure 10d,e). However, even at RH = 90%, when water molecules can reach *z* = 1.0 nm above the mica surface (Figure 7), hydration water does not reach a solid angle θ of less than 35°. Therefore, over the entire range of RHs studied here, contact-adsorbed CO_2_ molecules are not fully hydrated.

In this study, partial CO_2_ dissolution and carbonic acid formation, which could influence adsorption behavior, are neglected because the force fields used in our simulations do not account for chemical reactions. Developing or incorporating reactive force fields in future studies would be necessary to fully capture the interplay among CO_2_ dissolution, water adsorption, and potential chemical transformations, especially at RH higher than 90%.

#### 3.2.3 Quantitative Aspects of Water Adsorption

Figure 4b shows the effects of 100 bar of CO_2_ on the water adsorption isotherm, and Figure 6 shows the change of the areal density of each true interfacial water layer induced by 100 bar of CO_2_. (The water density profiles in the CO_2_-free and 100 bar of CO_2_ systems are presented side by side in Figure S1 to show their subtle differences, and their comparisons will be highlighted whenever necessary.) The modulation of water adsorption by 100 bar of CO_2_ can be classified into three stages.

In the first stage (RH = 0.01 to 20%), a significant (32.29%) reduction of adsorption occurs at RH = 0.01%. This reduction in adsorption becomes less significant as RH increases. The region in which water loss occurs shifts slightly away from the mica surface (Figure S1a–d). In the second stage (RH = 20 to 60%), only a minor reduction of adsorption occurs, and it is insensitive to the RH. In the last stage (RH = 60 to 90%), the water adsorption gradually switches from reduction to enhancement. At RH = 90%, a modest enhancement of water adsorption (7.26%) occurs. This enhancement is achieved by the addition of water molecules to the second and third true water layers on the mica surface (Figure 6) and occurs in the region *z* > 0.65 nm (Figure S1h).

These results suggest that high-pressure CO_2_ has a discernible effect on water adsorption at very low and high RHs, conditions that can occur in unsaturated tight media in applications such as CO_2_ sequestration and CO_2_-cushioned H_2_ storage in depleted unconventional reservoirs. To understand the above three stages of evolution, we note that for a mica surface in equilibrium with water vapor, introducing high-pressure CO_2_ in the environment modulates water adsorption through three key mechanisms.

First, CO_2_ can reduce water adsorption via two mechanisms: (1) it squeezes away water molecules from the water film on the mica, and (2) it disrupts water–water associations. The former is akin to the thinning of water film confined between two solid surfaces under the action of external pressures applied on those surfaces. In the latter, CO_2_ molecules penetrate between adsorbed water molecules to disrupt their association and thus suppress their adsorption. The film squeezing and water dissociation effects are countered by mica–water and water–water interactions, which for the water film thickness here (≲ 1 nm), can be called the hydration effects. Second, CO_2_ molecules can reduce water adsorption through surface displacement: because CO_2_ molecules also interact strongly with mica (see Figure 8c), they can displace some adsorbed water molecules. Third, direct CO_2_–water interactions can increase the level of water adsorption. van der Waals CO_2_–water interactions can lower the potential energy of interfacial water molecules to stabilize them energetically. Further, the collision with interfacial CO_2_ molecules affords water molecules at the liquid–vapor interface more freedom in orientation, thereby stabilizing them entropically. Although all three mechanisms can act in each stage, their importance varies with RH, leading to the overall effects of environmental CO_2_ on water adsorption as shown in Figure 4b.

In the first stage (RH < 20%), the reduction of water adsorption is contributed mainly by the first two mechanisms. In particular, CO_2_ surface displacement plays a critical role because, as shown in Figures 6 and S1a–d, the water loss at RH < 20% occurs via the removal of molecules in the region 0.21–0.4 nm, or equivalently, in the first true water layer on the mica surface (i.e., contact-adsorbed water). Such removal is consistent with the displacement of water from the mica surface by contact-adsorbed CO_2_ molecules. Indeed, Figure 8a shows that a notable amount of CO_2_ molecules exist on the mica surface up to an RH of 20%.

In the second stage (RH = 20–60%), the role of CO_2_ surface displacement in the reduction of water adsorption gradually diminishes as RH rises since CO_2_ contact adsorption vanishes rapidly (Figure 8a). The environmental CO_2_ is found to remove the water molecules in the tail region of the water density profile, which belongs to the second true water layer atop the mica surface (Figures S1e,f and 3). Such a water removal is mainly contributed to by the water film squeezing and disruption of water–water association by CO_2_. For example, at RH = 60%, the second true water layer already emerges, but molecules in this layer do not associate extensively with each other, leaving them poorly coordinated and susceptible to the removal by environmental CO_2_.

**Figure 11:**
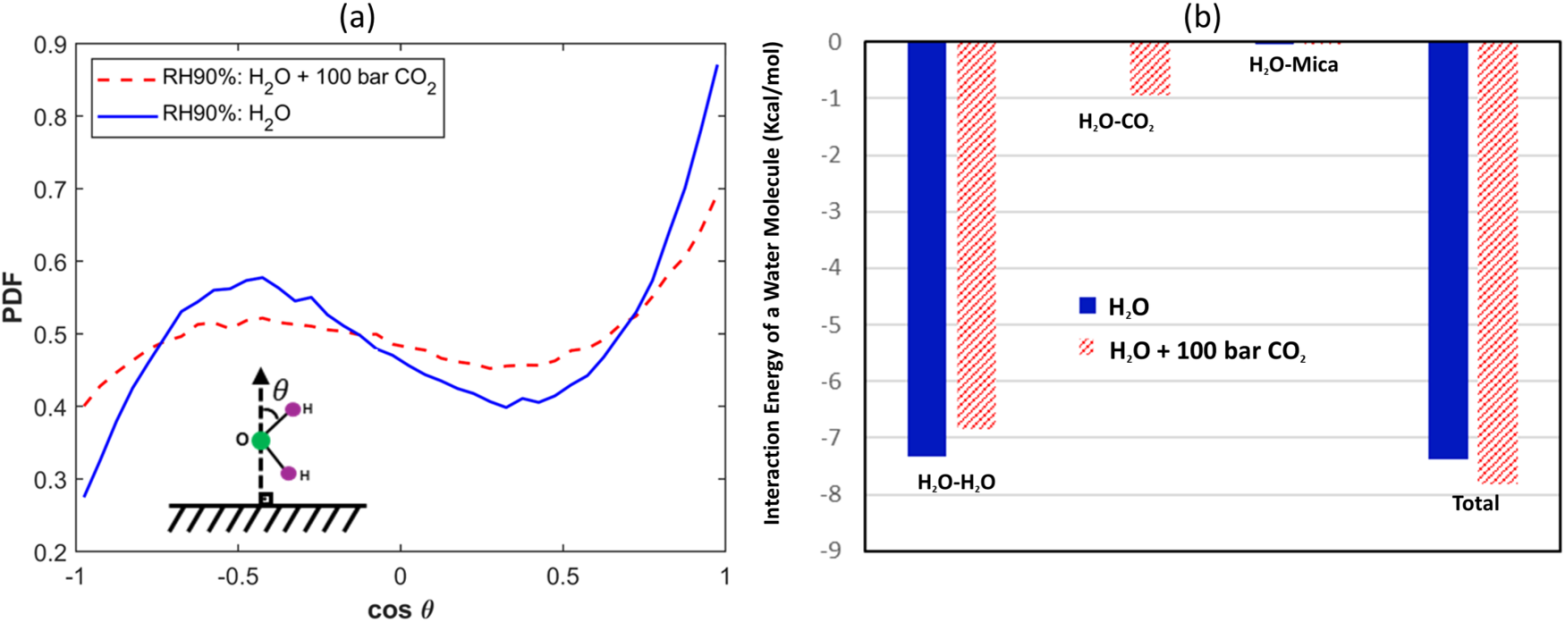
(a) Orientation distribution of the OH bond of water molecules located at the position where the water density is 5.65 nm^–3^ (cf. the filled black square in Figure 7). Solid (dashed) lines are for situations with a CO_2_ pressure of 0bar (100 bar). (b) The average interaction energy of water molecules located at the position where the water density is 5.65 nm^–3^.

In the final stage, as RH increases from 60 to 90%, the CO_2_ surface displacement no longer affects water adsorption. The weakening of the water–water association by CO_2_ still suppresses water adsorption. Meanwhile, the enhancement of water adsorption driven by direct CO_2_–water interactions becomes important near the water–vapor interface (or, near the tail of the water density profile, see Figure S1g,h). To appreciate this, we examine the orientation ordering and interaction energy of the water molecules located at positions where the mean water density is 5.65 nm^–3^ (cf. black squares in Figure 7 this position is selected as a representative position of water molecules directly exposed to the vapor phase (see Figure S4 and related text in the Supporting Information)). Figure 11a,b shows that introducing 100 bar of CO_2_ above the water film with RH = 90% makes the orientation of water molecules near the liquid–vapor interface more random, which favors water adsorption. Furthermore, 100 bar of CO_2_ atmosphere enhances molecules’ total potential energy near the interface by 0.42 kcal/mol, which also favors water adsorption. We note that introducing CO_2_ weakens water molecules’ potential energy contributed through water–water interactions by 0.50 kcal/mol, but this is compensated by the potential energy gain of 0.95 kcal/mol due to the appearance of CO_2_–water interactions (see Figure S5).

The suppression of water adsorption through the disruption of water–water association by CO_2_ becomes weaker as the RH increases. This is because, as RH increases, the second and third true water layers atop the mica surface become more developed, with molecules in them becoming associated with more water molecules. The latter makes the disruption of their associations by CO_2_ more difficult. On the other hand, the enhancement of water adsorption by direct CO_2_–water interactions is not as sensitive (see Figure S5). Consequently, their overall effect on water adsorption is negative at RH = 60%, becomes nearly zero at 80%, and is positive at RH = 90%.

## 4 Conclusions

The adsorption of pure CO_2_, water, and their mixture on mica surfaces under unsaturated conditions is investigated. Water adsorption at RH = 0.01–90% is found to be stronger than the CO_2_ adsorption at 100 bar. A subnanometer water film readily forms on the mica surface at relatively low RH, leading to apparent liquid–solid and liquid–vapor interfaces at RH = 0.01%. ITIM analysis reveals that the film is spatially heterogeneous, predominantly featuring patches of 1–3 layers of water molecules across the mica surface even at an RH of 90%. Because the first true water layer atop the mica is highly constrained and water molecules in further interfacial layers are not well coordinated with others at low RH, a true liquid–vapor interface likely emerges only as the RH approaches 60–80%. The coadsorption of CO_2_ and water is characterized by the strong suppression of CO_2_ contact adsorption. However, 100 bar of CO_2_ also lowers water adsorption by ∼30% at RH = 0.01% due to CO_2_’s surface displacement effect. This suppression is weakened as RH increases and water adsorption becomes enhanced by about 7% at RH = 90%. This transition is attributed to the diminished CO_2_ surface displacement, the weakening of CO_2_’s water film squeezing and water–water association disruption effects, and the stabilization of water at liquid–vapor interfaces by direct CO_2_–water interactions at high RH.

While the present study focuses on the CO_2_–water–mica system, its insights help understand the adsorption in other systems. The molecular structure of the heterogeneous water films on other mineral surfaces should show similar features as revealed here, and the true liquid–vapor interfaces in those systems are expected to emerge only at rather high relative humidities. Understanding such structures and interfaces aids in analyzing mass transfer at these interfaces (e.g., the value of the accommodation coefficient). The thermodynamics of the coadsorption of CO_2_ and water, especially the three mechanisms discussed above, can help understand the adsorption of multiple species on mineral surfaces. For example, given H_2_’s low density and its weaker interactions with mineral surfaces and water molecules, surface displacement, disruption of water–water association, and direct H_2_–water interactions all should be considerably weaker than the CO_2_ scenario studied here. Hence, we expect H_2_ to affect water adsorption on mineral surfaces only marginally. To what extent these expectations are true is of interest in the scenario of underground hydrogen storage, which may be explored in future studies.

## Supporting Information Available

Orientation distribution of the OH bond of water molecules; dynamics of water molecules in the first density peak; comparison of water density profiles near mica wall at different RH and CO_2_ pressures; the method for computing interaction energies; the method for selecting representative water molecules exposed to the vapor phase; and the interaction energy of water molecules located in the tail region of water density profiles (PDF)

## Notes

This article has been authored by UT-Battelle, LLC, under Contract DE-AC0500OR22725 with the U.S. Department of Energy. The United States Government retains and the publisher, by accepting the article for publication, acknowledges that the United States Government retains a nonexclusive, paid-up, irrevocable, worldwide license to publish or reproduce the published form of this manuscript, or allow others to do so, for the United States Government purposes. The Department of Energy will provide public access to these results of federally sponsored research in accordance with the DOE Public Access Plan (http://energy.gov/downloads/doe-public-access-plan).

## References

[ref1] Jia B.; Tsau J.-S.; Barati R. A review of the current progress of CO_2_ injection EOR and carbon storage in shale oil reservoirs. Fuel 2019, 236, 404–427. 10.1016/j.fuel.2018.08.103.

[ref2] Hawthorne S. B.; Miller D. J.; Jin L.; Azzolina N. A.; Hamling J. A.; Gorecki C. D. Lab and Reservoir Study of Produced Hydrocarbon Molecular Weight Selectivity during CO_2_ Enhanced Oil Recovery. Energy Fuels 2018, 32 (9), 9070–9080. 10.1021/acs.energyfuels.8b01645.

[ref3] Yu W.; Lashgari H. R.; Wu K.; Sepehrnoori K. CO_2_ injection for enhanced oil recovery in Bakken tight oil reservoirs. Fuel 2015, 159, 354–363. 10.1016/j.fuel.2015.06.092.

[ref4] Pang J.; Liang Y.; Masuda Y.; Matsuoka T.; Zhang Y.; Xue Z. Swelling Phenomena of the Nonswelling Clay Induced by CO_2_ and Water Cooperative Adsorption in Janus-Surface Micropores. Environ. Sci. Technol. 2020, 54 (9), 5767–5773. 10.1021/acs.est.0c00499. 32271553

[ref5] Qomi M. J. A.; Miller Q. R. S.; Zare S.; Schaef H. T.; Kaszuba J. P.; Rosso K. M. Molecular-scale mechanisms of CO_2_ mineralization in nanoscale interfacial water films. Nat. Rev. Chem. 2022, 6 (9), 598–613. 10.1038/s41570-022-00418-1. 37117714

[ref6] Liu Q.; Benitez M. D.; Xia Z.; Santamarina J. C. Pore-scale phenomena in carbon geological storage (Saline aquifers—Mineralization—Depleted oil reservoirs). Front. Energy Res. 2022, 10, 979573 10.3389/fenrg.2022.979573.

[ref7] Heinemann N.; Alcalde J.; Miocic J. M.; Hangx S. J. T.; Kallmeyer J.; Ostertag-Henning C.; Hassanpouryouzband A.; Thaysen E. M.; Strobel G. J.; Schmidt-Hattenberger C.; et al. Enabling large-scale hydrogen storage in porous media – the scientific challenges. Energy Environ. Sci. 2021, 14 (2), 853–864. 10.1039/D0EE03536J.

[ref8] Zivar D.; Kumar S.; Foroozesh J. Underground hydrogen storage: A comprehensive review. Int. J. Hydrogen Energy 2021, 46 (45), 23436–23462. 10.1016/j.ijhydene.2020.08.138.

[ref9] Pan B.; Yin X.; Ju Y.; Iglauer S. Underground hydrogen storage: Influencing parameters and future outlook. Adv. Colloid Interface Sci. 2021, 294, 102473 10.1016/j.cis.2021.102473. 34229179

[ref10] Li Y.; Nair A. K. N.; Kadoura A.; Yang Y.; Sun S. Molecular Simulation Study of Montmorillonite in Contact with Water. Ind. Eng. Chem. Res. 2019, 58 (3), 1396–1403. 10.1021/acs.iecr.8b05125.

[ref11] Chen Z.; Hu L. Adsorption behavior of benzene on clay mineral surfaces at different temperatures and air humidity based on molecular simulation. Appl. Clay Sci. 2023, 243, 107068 10.1016/j.clay.2023.107068.

[ref12] Chen G.; Lu S.; Zhang J.; Xue Q.; Han T.; Xue H.; Tian S.; Li J.; Xu C.; Pervukhina M. Keys to linking GCMC simulations and shale gas adsorption experiments. Fuel 2017, 199, 14–21. 10.1016/j.fuel.2017.02.063.

[ref13] Hui D.; Li L.; Zhang Y.; Peng X.; Li T.; Jia C.; Pan Y. Molecular simulation of adsorption behaviors of methane and carbon dioxide on typical clay minerals. Front. Energy Res. 2023, 11, 1231338 10.3389/fenrg.2023.1231338.

[ref14] Ali M.; Pan B.; Yekeen N.; Al-Anssari S.; Al-Anazi A.; Keshavarz A.; Iglauer S.; Hoteit H. Assessment of wettability and rock-fluid interfacial tension of caprock: Implications for hydrogen and carbon dioxide geo-storage. Int. J. Hydrogen Energy 2022, 47 (30), 14104–14120. 10.1016/j.ijhydene.2022.02.149.

[ref15] Peng F.; Xiong K.; Wang R.; Li Y.; Guo Z.; Feng G. Molecular Insight into Microbehaviors of n-Decane and CO_2_ in Mineral Nanopores. Energy Fuels 2020, 34 (3), 2925–2935. 10.1021/acs.energyfuels.9b04125.

[ref16] Wang R.; Peng F.; Song K.; Feng G.; Guo Z. Molecular dynamics study of interfacial properties in CO_2_ enhanced oil recovery. Fluid Phase Equilib. 2018, 467, 25–32. 10.1016/j.fluid.2018.03.022.

[ref17] Santos M. S.; Franco L. F. M.; Castier M.; Economou I. G. Molecular Dynamics Simulation of n-Alkanes and CO_2_ Confined by Calcite Nanopores. Energy Fuels 2018, 32 (2), 1934–1941. 10.1021/acs.energyfuels.7b02451.

[ref18] Moh D. Y.; Fang C.; Yin X.; Qiao R. Interfacial CO_2_-mediated nanoscale oil transport: from impediment to enhancement. Phys. Chem. Chem. Phys. 2020, 22 (40), 23057–23063. 10.1039/D0CP03930F. 33047766

[ref19] Du J.; Zhou A.; Zhong Y.; Shen S.-L. Molecular simulation on CO_2_ adsorption heterogeneity in montmorillonite nanopores with different surface charges in presence of water. Chem. Eng. J. 2024, 482, 148958 10.1016/j.cej.2024.148958.

[ref20] Jin Z.; Firoozabadi A. Methane and carbon dioxide adsorption in clay-like slit pores by Monte Carlo simulations. Fluid Phase Equilib. 2013, 360, 456–465. 10.1016/j.fluid.2013.09.047.

[ref21] Moh D. Y.; Zhang H.; Sun S.; Qiao R. Molecular anatomy and macroscopic behavior of oil extraction from nanopores by CO_2_ and CH_4_. Fuel 2022, 324, 124662 10.1016/j.fuel.2022.124662.

[ref22] Zhang W.; Feng Q.; Wang S.; Xing X.; Jin Z. CO_2_-regulated octane flow in calcite nanopores from molecular perspectives. Fuel 2021, 286, 119299 10.1016/j.fuel.2020.119299.

[ref23] Zhang H.; Wang S.; Wang X.; Qiao R. Enhanced Recovery of Oil Mixtures from Calcite Nanopores Facilitated by CO_2_ Injection. Energy Fuels 2024, 38 (6), 5172–5182. 10.1021/acs.energyfuels.3c05235. 38532839 PMC10961724

[ref24] Zhang W.; Dai C.; Chen Z.; He Y.; Wang S. Recovery mechanisms of shale oil by CO_2_ injection in organic and inorganic nanopores from molecular perspective. J. Mol. Liq. 2024, 398, 124276 10.1016/j.molliq.2024.124276.

[ref25] Malani A.; Ayappa K. G. Adsorption Isotherms of Water on Mica: Redistribution and Film Growth. J. Phys. Chem. B 2009, 113 (4), 1058–1067. 10.1021/jp805730p. 19123830

[ref26] Balmer T. E.; Christenson H. K.; Spencer N. D.; Heuberger M. The Effect of Surface Ions on Water Adsorption to Mica. Langmuir 2008, 24 (4), 1566–1569. 10.1021/la702391m. 18052225

[ref27] Cheng T.; Sun H. Adsorption of Ethanol Vapor on Mica Surface under Different Relative Humidities: A Molecular Simulation Study. J. Phys. Chem. C 2012, 116 (31), 16436–16446. 10.1021/jp3020595.

[ref28] Xia Y.; Cai M.; Wang Y.; Sun Q.; Dai Z. Competitive adsorption mechanisms of multicomponent gases in kaolinite under electric fields: A molecular perspective. Geoenergy Sci. Eng. 2024, 238, 212897 10.1016/j.geoen.2024.212897.

[ref29] Zhang M.; Jin Z. Molecular simulation on CO_2_ adsorption in partially water-saturated kaolinite nanopores in relation to carbon geological sequestration. Chem. Eng. J. 2022, 450, 138002 10.1016/j.cej.2022.138002.

[ref30] Kadoura A.; Nair A. K. N.; Sun S. Adsorption of carbon dioxide, methane, and their mixture by montmorillonite in the presence of water. Microporous Mesoporous Mater. 2016, 225, 331–341. 10.1016/j.micromeso.2016.01.010.

[ref31] Liu J. C.; Monson P. A. Does Water Condense in Carbon Pores. Langmuir 2005, 21 (22), 10219–10225. 10.1021/la0508902. 16229548

[ref32] Berendsen H. J. C.; Postma J. P. M.; van Gunsteren W. F.; Hermans J. Interaction Models for Water in Relation to Protein Hydration. In Intermolecular Forces; Pullman B., Ed.; The Jerusalem Symposia on Quantum Chemistry and Biochemistry; Springer : Netherlands, 1981; pp 331–342.

[ref33] Cygan R. T.; Liang J.-J.; Kalinichev A. G. Molecular Models of Hydroxide, Oxyhydroxide, and Clay Phases and the Development of a General Force Field. J. Phys. Chem. B 2004, 108 (4), 1255–1266. 10.1021/jp0363287.

[ref34] Zhu A.; Zhang X.; Liu Q.; Zhang Q. A Fully Flexible Potential Model for Carbon Dioxide. Chin. J. Chem. Eng. 2009, 17 (2), 268–272. 10.1016/S1004-9541(08)60204-9.

[ref35] Plimpton S. Fast Parallel Algorithms for Short-Range Molecular Dynamics. J. Comput. Phys. 1995, 117 (1), 1–19. 10.1006/jcph.1995.1039.

[ref36] Pártay L. B.; Hantal G.; Jedlovszky P.; Vincze Á.; Horvai G. A new method for determining the interfacial molecules and characterizing the surface roughness in computer simulations. Application to the liquid–vapor interface of water. J. Comput. Chem. 2008, 29 (6), 945–956. 10.1002/jcc.20852. 17963228

[ref37] Lbadaoui-Darvas M.; Idrissi A.; Jedlovszky P. Computer Simulation of the Surface of Aqueous Ionic and Surfactant Solutions. J. Phys. Chem. B 2022, 126 (4), 751–765. 10.1021/acs.jpcb.1c08553. 34904437 PMC9161821

[ref38] Fábián B.; Senćanski M. V.; Cvijetić I. N.; Jedlovszky P.; Horvai G. Dynamics of the Water Molecules at the Intrinsic Liquid Surface As Seen from Molecular Dynamics Simulation and Identification of Truly Interfacial Molecules Analysis. J. Phys. Chem. C 2016, 120 (16), 8578–8588. 10.1021/acs.jpcc.5b10370.

[ref39] Sega M.; Hantal G.; Fábián B.; Jedlovszky P. Pytim: A python package for the interfacial analysis of molecular simulations. J. Comput. Chem. 2018, 39 (25), 2118–2125. 10.1002/jcc.25384. 30306571 PMC6221047

[ref40] Michaud-Agrawal N.; Denning E. J.; Woolf T. B.; Beckstein O. MDAnalysis: A toolkit for the analysis of molecular dynamics simulations. J. Comput. Chem. 2011, 32 (10), 2319–2327. 10.1002/jcc.21787. 21500218 PMC3144279

[ref41] Jorge M.; Jedlovszky P.; Cordeiro M. N. D. S. A Critical Assessment of Methods for the Intrinsic Analysis of Liquid Interfaces. 1. Surface Site Distributions. J. Phys. Chem. C 2010, 114 (25), 11169–11179. 10.1021/jp101035r.

[ref42] Israelachvili J. N. Intermolecular and Surface Forces, 3rd ed.; Academic Press, 2011.

[ref43] Buckingham A. D.; Disch R. L.; Pople J. A. The quadrupole moment of the carbon dioxide molecule. Proc. R. Soc. London, Ser. A 1963, 273 (1353), 275–289. 10.1098/rspa.1963.0088.

[ref44] Malani A.; Ayappa K. G. Relaxation and jump dynamics of water at the mica interface. J. Chem. Phys. 2012, 136 (19), 194701 10.1063/1.4717710. 22612103

