# Supplementary material for: Molecular Structure and Thermodynamics of CO2 and Water Adsorption on Mica
Source: J Phys Chem B. 2025 Apr 24;129(18):4558–68. doi: 10.1021/acs.jpcb.5c01076 (PMC12067434; doi:10.1021/acs.jpcb.5c01076)
Supplement: Supplementary file 1 — jp5c01076_si_001.pdf [file jp5c01076_si_001.pdf]

Supporting Information

Molecular Structure and Thermodynamics of CO_2 and Water Adsorption on Mica

Mert Aybar,[1] Hongwei Zhang,[1] and Rui Qiao,[1,*] Jingsong Huang,[2] Bobby G. Sumpter,[2] Bicheng Yan,[3] and Shuyu Sun[3,4]

[1] Department of Mechanical Engineering, Virginia Tech, Blacksburg, VA 24061, United States

[2] Center for Nanophase Materials Sciences, Oak Ridge National Laboratory, Oak Ridge, TN 37831, United States

[3] Physical Science and Engineering Division, King Abdullah University of Science and Technology, Thuwal, 23955, Saudi Arabia

[4] School of Mathematical Sciences, Tongji University, Shanghai 200092, China

1. Density, orientation and dynamics of water molecules near mica surfaces

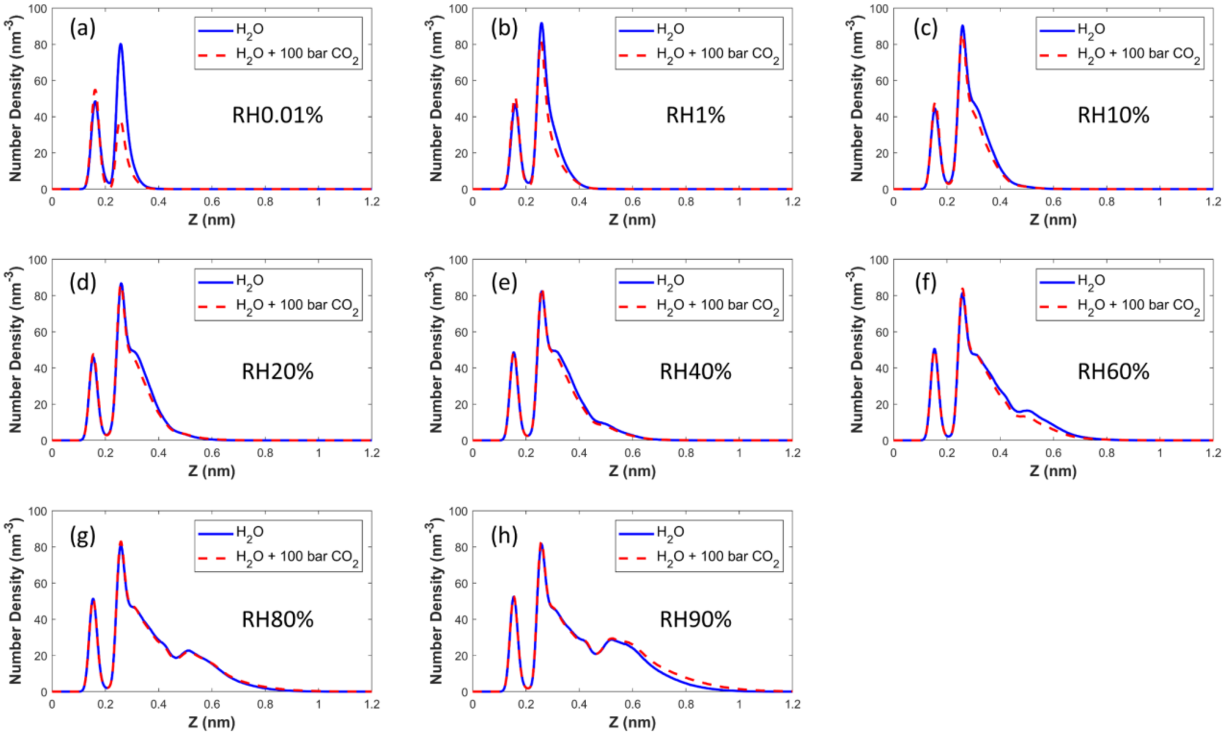

Fig. S1. Comparison of the water density profiles near the lower mica wall when the environmental CO_2 pressure is 0 bar and 100 bar at different RHs.

[*] To whom correspondence should be addressed. Email: ruiqiao@vt.edu

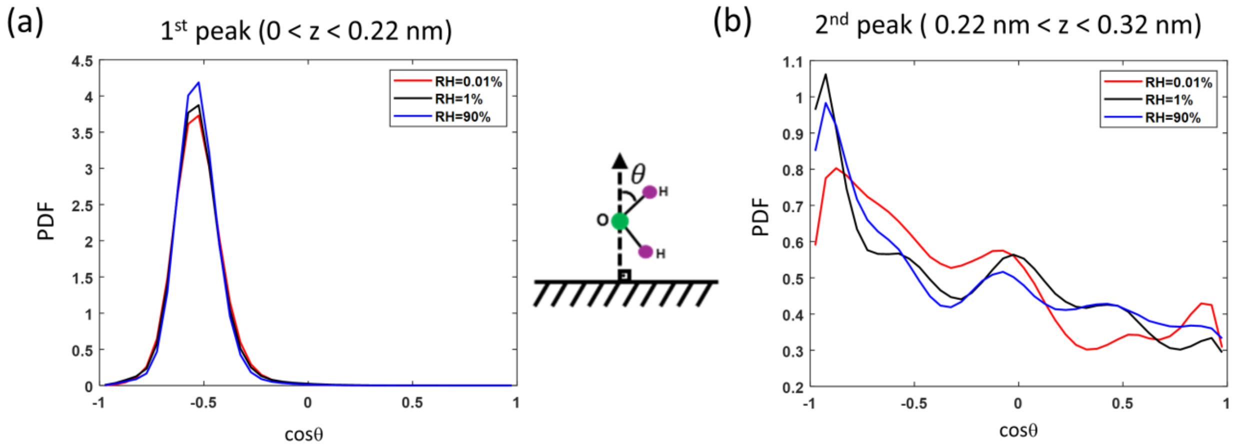

Fig. S2. Orientation distribution of the OH bond of water molecules in the regions $0 < z < 0.22$ nm (i.e., the first water peak at RH = 0.01%) and $0.22 \text{ nm} < z < 0.32$ nm.

To gain a basic understanding of dynamics of water adsorbed on mica, we compute the continuous survival probability (CSP) functions of the water molecules in the first density peak shown in Fig. 4a, as have been done by Malani and Ayappa.[1] In their work, a water slab of about 1.8 nm thick is confined between two mica sheets. CSP(t) measures the probability that water molecules reside continuously in the first density peak between 0 and time *t*. Figure S2a shows that the CSP of water molecules in the first density peak in these systems are essentially the same despite our water film is exposed to vapor but the water in Ref. 1 is confined between mica sheets. Such similarity may be attributed to the fact that water molecules in this peak occupy the ditrigonal cavities and form an ordered lattice on the mica surface,[1] and therefore their dynamics are governed by their interactions with mica. Malani and Ayappa showed that the CSP curve in Fig. S2a corresponds to a residence life time of about 15 ps, which is about 10 times larger than that for water molecules in bulk layers of a similar width with the first density peak. The sluggish dynamics revealed here suggest that the first water layer behave more like a solid rather than a liquid.

We also evaluated the dipole orientational correlation function ($C_{\mu,1}$) of the water molecules in the first density peak. As in Ref. 1, $C_{\mu,1}$ is defined as

$$C_{\mu,1}(t) = \frac{1}{\mathrm{CSP(t)}} \frac{\langle \sum_{i=1}^{N} P_1\big(\boldsymbol{\mu}_i(t) \cdot \boldsymbol{\mu}_i(0)\big) \cdot \prod_{t'=0}^{t} \Theta_i(t') \rangle}{\langle \sum_{i=1}^{N} P_1\big(\boldsymbol{\mu}_i(0) \cdot \boldsymbol{\mu}_i(0)\big) \cdot \Theta_i(0) \rangle}$$

where P_1 is the Legendre polynomial of rank 1, $\boldsymbol{\mu}_i(t)$ is the dipole vector of a water molecule *i*, and N is the number of water molecules. The function Θ_i is 1 if a water molecule *i* resides in the first density peak at time *t* and is otherwise *0*. Fig. S2b shows that $C_{\mu,1}$ of the water molecules in the first density

peak decays at a time scale larger than 10 ps, similar to that reported in Ref. 1. The slow decay of $C_{\mu,1}$ further supports the sluggish dynamics of water molecules in the first density peak.

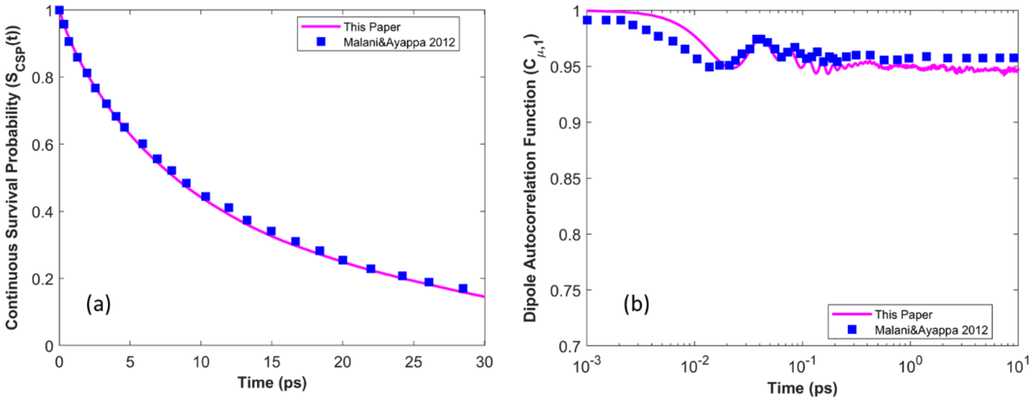

Fig. S3. A comparison of the continuous survival probability function (a) and the dipole orientational autocorrelation function (b) for water molecules in the first density peak near the mica surface obtained in this study and by Malani and Ayappa.[1] In Malania and Ayappa's work, water is confined between two mica sheets. In our data shown here, the RH of the water vapor above the mica is 0.01%.

2. Method for computing the interaction energies shown in Fig. 8c and 9

To compute the interaction energy between CO_2 and any species of interest (e.g., CO_2, mica, or H_2O molecules), LAMMPS' "rerun" command was used. For example, to determine components of CO_2's potential energy due to their interactions with mica, we loaded the trajectories saved during our original simulations but turned off the interactions between CO_2 and other species (CO_2 and H_2O) and used LAMMPS' "per/atom" command to evaluate CO_2-mica interactions for each CO_2 molecule in the system. Energy distribution can then be obtained through histograms. Documentations of LAMMPS' "rerun" and "pe/atom" commands can be found at https://docs.lammps.org/rerun.html and https://docs.lammps.org/compute_pe_atom.html

3. Selection of representative position for water molecules directly exposed to vapor

In the main text, the orientation and interaction energy of the water molecules directly exposed to vapor were analyzed. These molecules are selected to fall into a 0.04 nm-wide bin centering on the position where the mean water density is 5.65 nm^{-3}. The density of 5.65 nm^{-3} was selected based on the position of the third interfacial water layer (i.e., the water layer directly exposed to the vapor phase) at RH = 90%.

Specifically, using the ITIM analysis, we identified three layers of water molecules on the mica surface. For water molecules in each layer, we built a histogram based on their oxygen atoms' z-position, from which the probability density distribution was computed. Figure R3 shows that the probability density peaks at about z = 0.77 nm (marked by a black arrow), where the 1D water density

profile gives a value of 5.65 nm^{-3}. Therefore, it is reasonable to use water molecules at the location where the 1D density is 5.65 nm^{-3} to present the water directly exposed to the vapor phase.

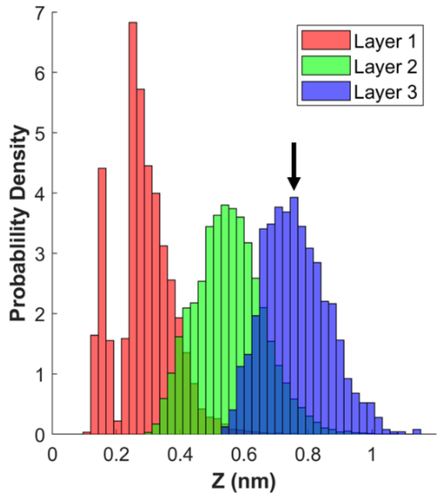

Fig. S4. The probability density distribution of the *z*-position of molecules in the three interfacial water layers on a mica surface at RH = 90%. To compute these distributions, for each saved trajectory frame, we first identify molecules belonging to the first, second, and third interfacial layers as discussed in the main text. Then, for water molecules in each of these layers, we build a histogram based on their oxygen atoms' z-position, from which the probability density distribution is computed.

4. Interaction energy of water molecules near the water-vapor interface

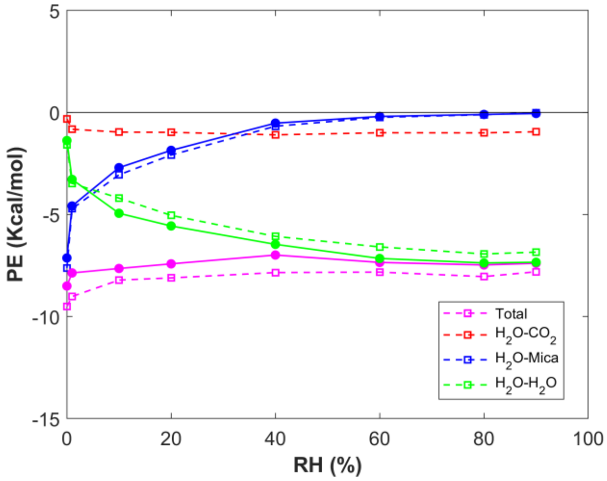

Fig. S5. The average interaction energy of water molecules located at the position where the water density is 5.65 nm^{-3}. Solid (dashed) lines are for situations with a CO_2 pressure of 0 bar (100 bar).

Reference

(1) Malani, A.; Ayappa, K. G. Relaxation and jump dynamics of water at the mica interface. *The Journal of Chemical Physics* **2012**, *136* (19), 194701.
